# Retraction

**DOI:** 10.1111/jcmm.16336

**Published:** 2021-03-14

Retraction: microRNA‐374 inhibits proliferation and promotes apoptosis of mouse melanoma cells by inactivating the Wnt signalling pathway through its effect on tyrosinase. Xiao‐Jing Li, Zhi‐Feng Li, Yan‐Yan Xu, Zhao Han and Zhi‐Jun Liu. *J Cell Mol Med*. 2019; 23: 4991– 5005 (https://doi.org/10.1111/jcmm.14348)

The above article from Journal of Cellular and Molecular Medicine, published online on 17 June 2019 on Wiley Online Library (wileyonlinelibrary.com) in Volume 23, pp. 4991‐5005, has been retracted by agreement between the authors, the journal Editor‐in‐Chief and John Wiley & Sons Ltd. The journal was informed by the authors of improper images used in Figure 1 and Figure 2, where tumour tissue images have mistakenly been included instead of paracancerous tissue. More follow‐up experiments are needed to confirm the validity of the results.

